# Deciphering the role of exosomal derived regulatory noncoding RNAs as potential biomarkers for diabetic retinopathy: a systematic review

**DOI:** 10.1186/s40942-024-00615-8

**Published:** 2024-12-18

**Authors:** Santosh Nandalal, Harshitha Venkatesan, Madhumitha Haridoss, Krithika Ramachandran, Raji Rajesh Lenin

**Affiliations:** Division of Medical Research, Faculty of Medicine and Health Sciences, SRM Medical College Hospital and Research Centre, SRM Institute of Science and Technology, Kattankulathur, Chengalpattu, Tamil Nadu 603203 India

**Keywords:** Diabetic retinopathy, Exosomes, noncoding RNAs, miRNA, Circular RNA, Inflammation, Angiogenesis, Systematic review

## Abstract

About one-third of individuals with diabetes develop diabetic retinopathy (DR), with one-tenth experiencing vision-threatening conditions such as diabetic macular edema (DME) or proliferative diabetic retinopathy (PDR). Current treatments only show recovery in 50% of cases, and the disease often remains asymptomatic. Therefore, novel early detection methods and new biomarkers are crucial. Our study aims to summarise evidence on the role of altered exosomal regulatory noncoding RNAs in DR, based on human studies. Observational studies delineating the different exosomal noncoding RNAs in DR were searched systematically from inception to June 2024 in PubMed-MEDLINE and SCOPUS databases. Preferred Reporting Items of Systematic Reviews and Meta-analysis (PRISMA) 2020 guidelines was used to conduct this systematic review (Prospero ID: CRD42023406724). We synthesised data from 13 human studies that assessed the expression of exosomal regulatory ncRNAs from individuals with DR. Among the 13 studies, nine focused on miRNA, while three examined lncRNA and one on circRNA. Although no common genes were identified across the studies, the review underscores the critical roles of exosomal noncoding RNAs in the pathogenesis of diabetic retinopathy, highlighting their potential as therapeutic targets. Future studies should aim for a unified approach in terms of sample selection, RNA analysis, and appropriate controls for a comprehension of exosomal expression patterns for early diagnosis of DR.

**Supplementary Information:**

The online version contains supplementary material available at 10.1186/s40942-024-00615-8.

## Introduction

Diabetic Retinopathy (DR) is a vision-threatening complication of Diabetes Mellitus (DM) [1]. It causes damage to the blood vessels of the light-sensitive tissues of the eye and could lead to severe eye complications like vitreous haemorrhage, retinal detachment, glaucoma, and blindness [2]. 191 million people are expected to have DR by 2030, which could potentially result in blindness [3]. The asymptomatic nature of the disease in its early stages calls for novel detection methods [4]. While treatments like anti-Vascular Endothelial Growth Factor (anti-VEGF) drugs and laser photocoagulation can reduce vision loss in severe DR, early-stage disease management remains crucial [5]. The escalating prevalence of DR has highlighted the inadequacies of existing treatment modalities, as successful restoration of vision is limited to a mere 40–50% of patients [6, 7]. Adding to it, the disease being asymptomatic until the pathology is significantly advanced, novel approaches to detect it during the early stages are necessary. As the treatment outcomes of this chronic disease are still unsatisfactory, identifying preventive measures and new biomarkers for the early detection of disease is the need of the hour [8].

In this regard, circulating biomarkers could be potentially used for early diagnosis, to identify diabetic patients who are most prone to progressive worsening and require regular follow up [9]. In recent years, noncoding RNAs (ncRNAs) have emerged as novel biomarkers due to their pivotal role in the development and progression of DR [10, 11]. ncRNAs make up a large portion of transcriptome and are functional RNA molecules lacking protein-coding potentials [12]. They regulate gene expression by interacting with proteins and other RNA molecules, affecting various cellular processes such as transcription, translation, and chromatin remodelling. They often serve as scaffolds, bringing protein complexes to specific genomic regions to control gene activity. Depending on the type of ncRNA, their mechanisms include acting as decoys, guiding chromatin modifications, and inhibiting mRNA translation through base pairing [13]. These ncRNAs, such as microRNAs (miRNAs), long noncoding RNAs (lncRNAs), and circular RNAs (circRNAs), play a crucial role in regulating gene expression and impact various biological processes associated with retinopathy. To uncover new DR biomarkers, researchers have conducted expression profiling studies on lncRNAs, circRNAs, and miRNAs in cell lines, animal models, and human samples [14, 15].

Studies highlight exosomes, tiny endocytic vesicles released by most cells, carry a diverse array of RNA that can alter how recipient cells behave, potentially serving as disease biomarkers [16]. Studies highlight exosomes as a better source of ncRNA for biomarker profiling compared to non-exosomal or unfractionated samples, especially in miRNA-scarce samples like the vitreous [17]. Additionally, exosome content reflects the parent cells’ metabolic state and offers valuable insights into intercellular communication [18]. Recent studies have suggested that lncRNAs, circRNAs and miRNAs compete with each other through miRNA response elements (MREs) and modulate the progression of diabetes, which may function as miRNA sponges [19].

Literature till date examining the expression of exosomal ncRNAs in humans have shown considerable heterogeneity in sample types, control groups along with limited sample size creating a substantial research gap. Therefore, a systematic review of these studies is crucial to collectively assess the individual study findings to understand how ncRNAs modulate key cellular processes, such as inflammation, angiogenesis, oxidative stress, and apoptosis. Further this will enable us to draw cohesive conclusions for deciphering the potential of exosomal ncRNA to serve as biomarkers for DR screening.

## Methods

### Search strategy and selection criteria

The present systematic review was conducted adhering to the preferred reporting items of systematic reviews and meta-analysis (PRISMA) 2020 guidelines [20], and the protocol was registered at PROSPERO (CRD42023406724). Observational studies delineating different noncoding RNAs in DR were systematically searched in PubMed-MEDLINE and SCOPUS databases from inception till 25 June 2024. The search strategy was constructed using the search terms relevant to Problem (“Diabetic retinopathy” OR “Diabetic macular edema” OR “Proliferative Diabetic Retinopathy”) AND Outcome (exosome OR “multivesicular bodies” OR “intraluminal vesicles”) AND (ncRNA OR microRNA OR circularRNA OR long non-coding RNA) The detailed search strategies for Pubmed and Scopus are provided in Supplementary Tables 1 and 2 respectively. No restrictions based on languages or publication dates were imposed on the searches.

### Eligibility criteria

Observational studies that compared the expression of exosomal ncRNAs (miRNA, cirRNA and lncRNA) in DR with Type 2 Diabetes Mellitus (T2DM) alone or healthy subjects in any sample type (human plasma, serum, tears, and vitreous) were included in the review. Studies that provided detailed data on exosome isolation and quantification along with rigorous analysis of non-coding RNAs (ncRNAs) only were included.

Exclusion criteria included studies reporting exosomal RNAs and gene regulation in Type 1 Diabetes (T1D), because of it’s an early-onset condition and is typically symptomatic. Patients diagnosed with Type 1 Diabetes are routinely screened for DR starting five years after diagnosis. In contrast, Type 2 Diabetes is often asymptomatic, and many patients are already at risk of or have developed DR by the time of diagnosis. This difference in screening practices and disease progression informed our decision to focus on Type 2 Diabetes in this study. T2DM without retinopathy or other diabetic complications were excluded. Interventional studies and studies conducted in animal or cell models were excluded. Studies not having sufficient data for analysis even after contacting the author were also excluded. Reviews, case reports, newspaper articles, editorials, commentaries, book chapters were excluded. Studies published in language which neither reviewers nor translation applications are able to translate were excluded.

### Study screening and selection

Screening of studies identified in search was conducted using a Rayyan-web based tool [21]. Two reviewers (RRL and HM) screened the title and abstract (TiAb) independently after duplicate removal. Further, full-texts of the studies included in TiAb screening were reviewed independently by two reviewers (RRL and SN). Disagreements between two reviewers were resolved by consensus with the third reviewer.

### Data extraction and analysis

Data extraction form was created in MS Excel 2013 and data extraction was performed independently by SN and HV. Data on general study information, study characteristics, characteristics of studied population, details of methods and outcomes etc. were extracted. In the general information domain, author, title, journal, year of publication, contact of author were collected. In the study characteristics domain, study’s country, perspective, funding and conflict of interest were collected. In the characteristics of the studied population domain, details of the study population such as age, gender, diabetes status were collected. In the methods domain, exosome isolation, type of biological samples, platform, or method of ncRNA expression profiling were collected. In the outcomes data domain, a list of up regulated and down regulated ncRNAs and the pathways involved were recorded.

### Risk of bias assessment

Risk of bias assessment for the included studies was performed using the Newcastle- Ottawa Scale (NOS) for case-control studies. The NOS covered aspects like the risk of bias for subject selection, comparability, and the assessment of outcome/exposure in case-control studies. A study with a score ≥ 7 is considered to be of high quality, although this is not a standard criterion [22]. After risk of bias assessment, the overall rating of “Low risk,” “Moderate risk,” or “High risk” was given to each study. The “Low risk” rate is considered to be least biased whereas the “High risk” rate indicates significant bias which might invalidate the results [23]. The quality of included studies was appraised independently by the reviewers (HV and SN), and the disagreements were addressed by mutual consensus or with the intervention of a third reviewer.

## Results

### Studies identified by database searches

**Fig. 1:**
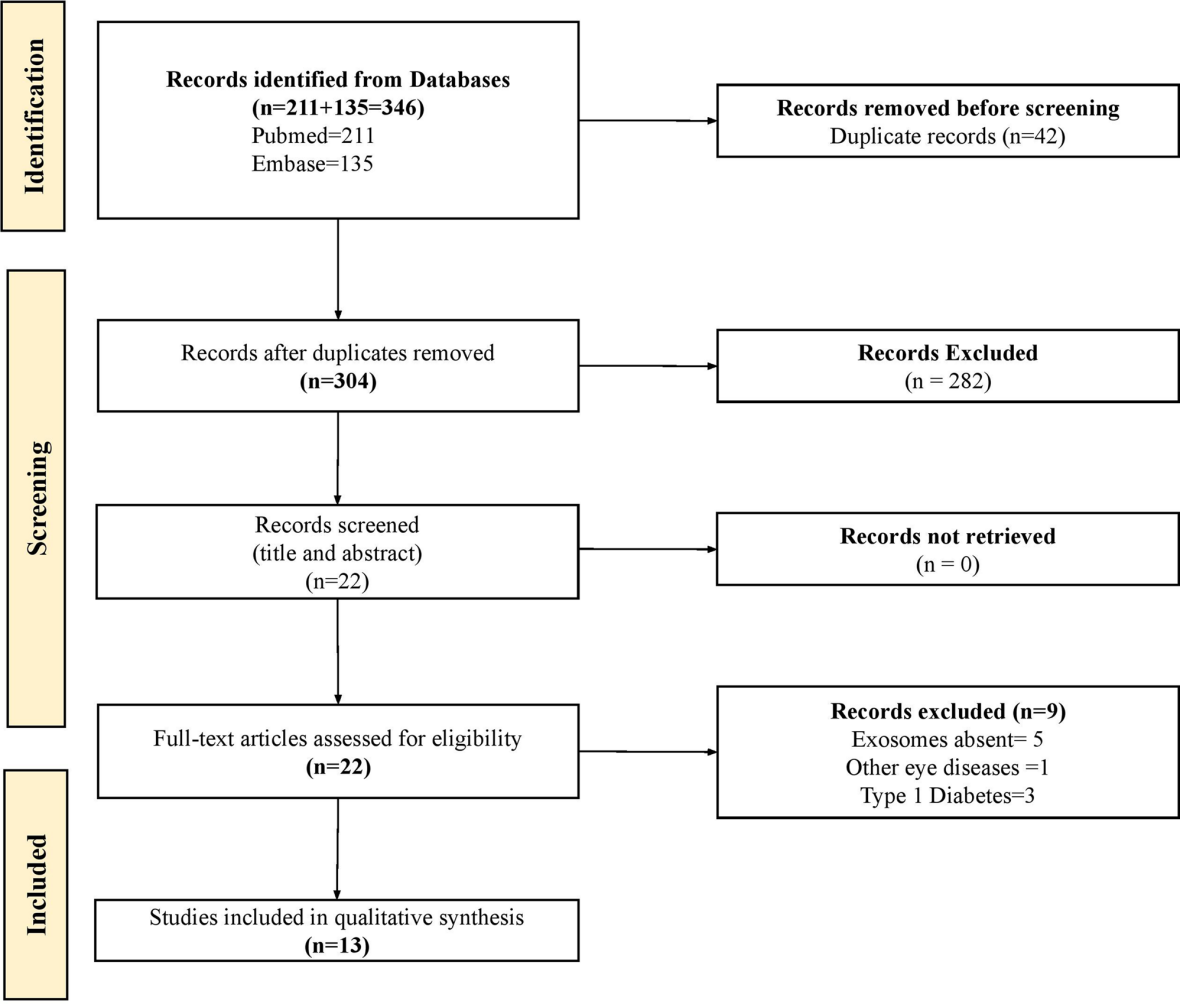
Flowchart depicting the study selection process using the PRISMA guidelines. This figure visually outlines the inclusion and exclusion of articles at different stages of the review

Our systematic search identified a total number of 311 studies. After removing duplicates, the title and abstract of 304 studies were reviewed. Of these, 282 were excluded due to irrelevance to our study or inclusion of non-diabetic subjects. Consequently, 23 articles were selected for full-text screening. Out of these, 13 articles met the eligibility criteria and were included in this systematic review. The remaining 9 articles were excluded due to reasons such as incorrect population (T1D), absence of exosome-related content or unavailability of full-text. The PRISMA flow chart elucidating the study selection process is represented in Fig. 1.

All the studies included were case-control studies with sample sizes ranging from 18 to 226 participants. The sample size for the study by Yu et al. (2021) could not be obtained despite efforts to contact the author. Nine studies reported differentially expressed exosomal miRNAs [24–32], three studies focused on altered lncRNAs [15, 33, 34], and one study examined dysregulated circRNAs [35]. RNA extraction samples varied across studies: vitreous humour was used in four studies [15, 26, 27, 33], blood plasma in three studies [7, 28, 32], blood serum in four studies [25, 30, 31, 35] and tear samples in two studies [24, 29].

All studies compared the DR patients with T2DM patients, except for two studies. Kot and Kaczmarek, 2022 compared the PDR with Macular hole [26] and Li et al. 2021 compared the PDR group with the senile cataract group [35]. Table 1 summarises the characteristics of the 13 included studies. Methodological aspects of the study including exosome isolation, RNA isolation and quantification techniques are summarised in Table 2.

Finally, we have also comprehensively synthesised the data from these studies and tabulated the key findings from these studies providing its clinical relevance (Table 3).

**Table 1:** Characteristics of included studies

Study (Year)	Type of RNAs	Sample	Control (Sample size)	Cases (Sample size)	Reference
Wan et al. (2017)	miRNA	Serum	HC (74), T2DM (76)	T2DMC (76) [in which DR (29)]	[30]
Jiang et al. (2021)	miRNA	Serum	T2DM (20)	DME (24)	[25]
Yu et al. (2021)	miRNA	Plasma	HC, DM	NPDR, PDR	[32]
Liu et al. (2021)	miRNA	Vitreous humor	MH (15)	PDR (10)	[27]
Santovito et al. (2021)	miRNA	Plasma	DM (10), HC (10)	DR (20)	[28]
Kot and Kaczmarek (2022)	miRNA	Vitreous humour	T2DM and MH (10)	PDR (10)	[26]
Hu et al. (2022)	miRNA	Tear	HC(11), DM(10)	DR(9)	[24]
Yang et al. (2023)	miRNA	Serum	DM (9)	DR (9)	[31]
Torimura et al. (2023)	miRNAs	Tear	HC (14)	AMD (8), NPDR(4), PDR(9)	[29]
Ye et al. (2022)	lncRNA	Plasma	T2DM (62)	DR(62)	[34]
Hu et al. (2023)	lncRNA	Vitreous humor	MH (40)	PDR (25)	[14]
Li et at (2024)	lncRNA	Vitreous humor	MH(50)	PDR(50)	[33]
Li et al. (2021)	CircRNA	Serum	Senile Cataract (10)	DM-PDR (10)	[35]

Legend HC - Healthy Controls, T2DM - Type 2 Diabetes Mellitus, T2DMC - Type 2 Diabetes Mellitus with Microvascular Complications, DME - Diabetic Macular Edema, DR - Diabetic Retinopathy, PDR- Proliferative Diabetic Retinopathy, NPDR - Non-Proliferative Diabetic Retinopathy, MH - Macular Hole, AMD - Age-related Macular Degeneration

### Studies characterising exosomal microRNA modulations and their impact on DR progression

Among the included studies, nine focussed on miRNA expression profiles in DR. Wan et al. initiated this exploration by identifying increased serum miR-7 in T2DM and its microvascular complications including DR [30]. The study revealed that circulating miR-7 predominantly existed in an exosome-free form rather than within membrane-bound exosomes which targets multiple components of the mTOR signaling pathway.

Jiang et al. elucidated the angiogenic role of miR-377-3p by showing its negative regulation of VEGF expression [25]. Yu et al. observed that miR-431-5p among the top four differentially expressed miRNAs (miR-431-5p, miR-142-5p, miR-361-3p and miR-181 d-5p) as a significant regulator associated with MAPK, RAP1, and RAS signalling pathways [36]. Their KEGG pathway analysis linked miR-431-5p to processes such as cell adhesion, cAMP signalling, and glucose metabolism, demonstrating the broad impact of miRNA on cellular functions relevant to DR. Overall, the study revealed that MiR-431-5p exhibited higher expression levels in small extracellular vesicles (sEVs) derived from patients with diabetic retinopathy (DR) as opposed to circulating miRNAs in DR patients.

Liu et al. further demonstrated that miR-9-3p among the top five upregulated miRNAs (miR-9-3p, miR-6511b-5p, miR-1285-3p, miR-505-5p, miR-4685-3p) activates VEGFR2 signalling pathway through the S1P1 axis, both in the presence and absence of exogenous VEGF-A [27]. Similar findings of aberrated miRNA expressions were described by Kot and Kaczmarek [26]. They identified 26 differentially expressed miRNAs, including 16 downregulated (miR-125a-5p, miR-125b-5p, miR-204-5p, miR-412-3p, miR-137, miR-361-3p, miR-211-3p, miR-9-3p, miR-30e-3p, miR-375, miR-9-5p, miR-30a-5p, miR-328-3p, miR-345-5p, miR-100-5p, and miR-543-3p) and 10 upregulated ones (miR-21-5p, let-7 g-5p, miR-660-5p, miR-142-3p, miR-19a-3p, miR-142-5p, miR-15a-5p, miR-103a-3p, miR-92a-3p, and miR-16-5p) between PDR and controls. Through experimental validation, they provided strong evidence linking miR-125 family, miR-204-5p, miR-21-5p, miR-41-3p, and let-7 g-5p 2 to mechanisms driving fibrovascular membrane development in PDR, affecting processes like angiogenesis, inflammation, and tissue remodelling. Notably, miR-21-5p upregulation, and the miR-125 family downregulation played a significant role in altering the TGF-β and VEGF signalling pathways. These studies emphasise the role of miRNAs in modulating angiogenic factors via VEGF signalling pathway which is pivotal in DR progression.

Another study conducted by Santovito et al., identified 12 upregulated miRNAs (let-7a-5p, miR-16-5p, miR-23a-3p, miR-25a-3p, miR-27a-3p, miR-92a-3p, miR-150-5p, miR-197-3p, miR-223-3p, miR-320a-3p, miR-320b, miR-486-5p) and 2 down regulated miRNAs (miR-346 and miR-495-3p) between DR and T2DM [28]. Further experimental validation downstream highlighted upregulation of miR-23a-3p, miR-25-3p and miR-320b, alongside downregulation of miR-495-3p leading to angiogenesis through NOTCH1 and β-Catenin pathways. Hu et al. identified several dysregulated miRNAs, including upregulated miR-145-5p, miR-214-3p, miR-9-5p, and miR-218-5p, as well as downregulated miR-146a-5p, miR-31-5p, and miR-96-5p [24]. KEGG pathway analysis connected these miRNAs to energy metabolism, insulin secretion, and resistance, including ErbB signaling, highlighting their widespread impact on metabolic and signaling pathways in DR.

In a study by Yang et al. among 18 differentially expressed miRNAs, four miRNAs (miRNA-3976, PC-5p-39533, and PC-3p-37421) showed a larger fold change [31]. The study highlighted in particular that, miRNA-3976 overexpression inversely correlates with NFκB, suggesting a protective role in regulating retinal ganglial cells (RGC) proliferation and apoptosis, adding another layer to the miRNA-mediated regulation of cell survival in DR. Torimura et al. in a pilot study identified higher expression of miR-151-5p in AMD and miR-422a in DME, although their specific pathways remain unspecified, indicating potential new areas for further research [29].

Though observations from the above investigated studies signifies the potential role of exosomal miRNAs in DR pathogenesis, there is no consistent pattern of dysregulation of specific miRNAs across all the studies.

### Studies characterising exosomal lncRNA modulations and their impact on DR progression

Three of the included studies investigated the role of exosomal lncRNA in DR. Ye et al. identified exosomal Distal-less homeobox 6 antisense 1 (DLX6-AS1), and Aminoacylase-1 (ACY1), and Rho GTPase activating protein (ARHGAP), which was overexpressed and regulating the p38–MAPK pathway [34]. They also found Psoriasis-susceptibility-related RNA gene induced by stress (PRINS) and Family with Sequence similarity 190, Member A3 (FAM190A-3), which was modulating the TGF-β signalling pathway to be underexpressed in DR.

In another study, Hu et al. were the first to report that lncRNA, LOC100132249 in vitreous samples from PDR patients, mediated endothelial dysfunction through the Wnt/β-Catenin signaling pathway [15]. The study revealed the crucial role of LOC100132249/miR-199a-5p/SNAI1 axis in endothelial-to-mesenchymal transition causing endothelial dysfunction. Lastly, Li et al. focused on the role of exosomal lncRNA-MIAT in DR, demonstrating that it modulates the MMP-X1 expression pathway by sponging miR-133a-3p [33]. This regulation influences tube formation, migration, and proliferation, thereby affecting retinal neovascularization. Their findings highlight how lncRNA-MIAT contributes to angiogenesis and the formation of new blood vessels in the retina, a critical aspect of DR progression. Together, these studies illustrate a complex interplay of ncRNAs-DLX6-AS1, PRINS, LOC100132249, miR-199a-5p, and lncRNA-MIAT that regulate various signalling pathways such as TGF-β, Wnt/β-Catenin, and VEGF.

### Studies exhibiting variations in exosomal circRNA profiles: insights into mechanisms and applications

Only one among the included studies examined circRNAs expression pattern in DR. circRNAs are widely expressed in mammalian serum and serve as a sponge for miRNAs to regulate gene expression [36, 37]. Li et al. 2021 identified 26 exosomal circRNAs in serum specimens [35]. Further they constructed a Competing endogenous RNA (ceRNA) network involving circRNA–miRNA–mRNA exosomal circRNA from the patients with DR. They observed that circFndc3b and circFAM13B are upregulated in DR. These circRNAs regulate PI3K-AKT, RAS, and MAPK signalling pathways, promoting angiogenesis and reducing cardiomyocyte apoptosis and fibrosis.

Abnormal angiogenesis, a benchmark in the development of DR, can disrupt the blood-retinal barrier and retinal microenvironment [38]. From all the above investigated studies, we have strong evidence that exosomal ncRNAs play an important role in angiogenesis and are expressed through the VEGF signaling pathway. Its effects are executed through a cascade of multiple signaling pathways such as mTOR, MAPK, Wnt/β-catenin, NF-κB, TGF-β signaling pathways, which are involved in the angiogenesis, cell growth and proliferation.

Together, these studies depict a comprehensive network of exosomal ncRNAs intricately involved in various signaling pathways, elucidating their critical roles in the pathogenesis of diabetic retinopathy.

### Studies with differentially expressed miRNAs across the studies

To identify potential common ncRNAs among the studies, we conducted a systematic comparison of the differentially expressed ncRNAs reported in each study. Across the 13 included studies, a total of 35 differentially expressed ncRNAs were identified. These were categorized based on their upregulation or downregulation compared to control groups, as summarized in Table 3.

Despite this comprehensive comparison, no single ncRNA was found to be consistently differentially expressed across multiple studies. This lack of commonly expressed ncRNAs across studies highlights the variability due to the potential differences in experimental conditions, methodologies, or samples used among the studies. These inconsistencies underscore the need for further research as there are no specific ncRNAs that are universally differentially expressed to identify strong biomarkers for diabetic retinopathy.

**Table 2:** Techniques of exosomal ncRNAs isolation and quantification

Study (year)	Exosomes- Isolation technique	ncRNA- Isolation technique	ncRNA Quantification	Reference
Wan et al. (2017)	Total Exosome Isolation Kits	Trizol Method	qRT-PCR TaqMan miRNA probes	[30]
Jiang et al. (2021)	ExoQuick Exosome Precipitation Solution	RNA extraction kit	qRT-PCR	[25]
Yu et al. (2021)	Sequential Differential centrifugation	miRNeasy Serum /Plasma Kit	miScript II RT Kit	[32]
Liu et al. (2021)	RiboTM Exosome Isolation Reagent	HiPure Liquid miRNA Kit	NA	[27]
Santovito et al. (2021)	ExoQuick exosome precipitation solution	miRNeasy kit	qRT-PCR	[28]
Kot and Kaczmarek (2022)	miRCURY Exosome Isolation Kit	miRNeasy Mini Kit	miRCURY LNA RT Kit	[26]
Hu et al. (2022)	Two-step Centrifugation	NA	qRT-PCR	[24]
Yang et al. (2023)	Ultracentrifugation	miRNeasy Micro Kit	qRT-PCR	[31]
Torimura et al. (2023)	ExoQuick-TC	SeraMir Exosome Amplification kit	qRT-PCR	[29]
Ye et al. (2022)	ExoQuick exosome precipitation solution	miRNeasy Micr Kit	qRT-PCR	[34]
Hu et al. (2023)	Multi-step Centrifugation	Ribo Exosome Isolation reagent	NA	[14]
Li et al. (2024)	Total Exosome Isolation Kits	Trizol Method	qRT-PCR	[33]
Li et al. (2021)	Total Exosome Isolation Kits	Trizol Method	ND-1000 NanoDrop	[35]

**Table 3:** A comprehensive study synthesis on the exploration of included studies: Key findings and clinical relevance

Study (year)	Upregulated genes in DR	Downregulated genes in DR	Cell Signalling Pathway	Key findings	Reference
Wan et al. (2017)	miR-7	NA	mTOR	Increased serum miR-7 are significantly involved in targeting multiple components of the mTOR signalling leading to b-cell dysfunction and insulin secretion.	[30]
Jiang et al. (2021)	NA	miR-377-3p	VEGF expression	miR-377-3p negatively regulates VEGF expression by directly binding to it	[25]
Yu et al. (2021)	miR-431-5p,	NA	MAPK, RAP1, RAS	KEGG pathway analysis revealed the target genes of miR-431-5p were associated with cell adhesion molecules pathway, cAMP signalling pathway, Rap1 signalling pathway and oocyte meiosis, while GO analysis showed the target genes involved in biological process include negative regulation of JAK-STAT cascade, positive regulation of glucose metabolic process.	[32]
Liu et al. (2021)	miR-9-3p, miR-9-3p, miR-6511b-5p, miR-1285-3p, miR-505-5p, miR-4685-3p	NA	VEGFR2 S1P_1_ - AKT/ERK	miR-9-3p/S1p_1_ axis promotes angiogenesis by activating the VEGFR2 signalling pathway in the presence or absence of exogenous VEGF-A.	[27]
Santovito et al. (2021)	miR-25-3p, miR-320b,, miR-16-5p,	miR-495-3p,	NOTCH1, β-Catenin	REACTOME database identified a significant enrichment in process relevant for endothelial proliferation, such as the regulation of the NOTCH1, β-Catenin pathways, while GO analysis revealed these targets to be involved in crucial pathways including regulation of metabolic process of cell response to stress, and development of blood vessels (angiogenesis).	[28]
Kot and Kaczmarek (2022)	miR-21- 5p, let-7 g-5p	miR-125a-5p, miR-125b-5p, miR-204-5p, miR-412-3p	TGF-β and VEGF	The expressions of these miRNAs regulate the multiple signalling pathways and can simultaneously target various angiogenic factors, including TGF-β and VEGF.	[26]
Hu et al. (2022)	miR-145-5p, miR-214-3p, miR-9-5p, and miR-218-5p	miR-146a-5p, miR-31-5p, miR-96-5p	ErbB	The KEGG pathway identified some common biological pathways, of these dysregulated miRNAs in both DM and DR such as energy metabolism (fatty acid metabolism, N-glycan biosynthesis, proteoglycans), insulin secretion (hippo signalling), and insulin resistance (ErbB signalling).	[24]
Yang et al. (2023)	miRNA-3976	NA	NFκB	The overexpression of miRNA is indirectly proportional to NFκB, and proper content of miRNA-3976 could partly exert a protective role while excess of miRNA-3976 could regulate multiple pathways leading to RGC proliferation and reduce apoptosis.	[31]
Torimura et al. (2023)	miR-151-5p, miR422a	NA	NA	Identified higher expression of miR-151-5p in AMD and miR-422a in PDR with DME.	[29]
Ye et al. (2022)	DLX6-AS1	PRINS	TGF-β	These dysregulated miRNAs can affect the TGF-β signalling pathway causing Endothelial dysfunction, neurodegeneration, and vascular damage. Furthermore, exosomal DLX-AS1 was only associated in DR with male, whereas exosomal PRINS was associated with DR in both male and female.	[34]
Hu et al. (2023)	LOC100132249	NA	Wnt/β-Catenin	The elevated exosomal miRNA influences endothelial dysfunction by means of the miR-199a-5p/SNAI1 axis, leading to the activation of Wnt/β-Catenin signaling pathways. It was observed that the LOC100132249/miR-199a-5p/SNAI1 loop regulates EndMT.	[14]
Li et al. (2024)	lncRNA-MIAT	NA	VEGF	By modulating the MMP-X1 expression pathway through sponging miR-133a-3p, exosomal lncRNA-MIAT can regulate tube formation, migration, proliferation and retinal neovascularization.	[33]
Li et al. (2021)	circFndc3b circFAM13B	NA	PI3K-AKT, RAS and MAPK	The upregulation of these miRNAs are found to facilitate angiogenesis *in vitro.*It was found to reduce cardiomyocyte apoptosis and fibrosis and enhance angiogenesis by promoting VEGF in the cardiovascular system. However, in the retina, it’s abnormal, with limited capacity to perform normal physical functions and high possibility of leakage.	[35]

(NA- Not available)

Legend: Upregulated or downregulated in comparison to the expression levels of these ncRNAs in controls

### Risk of bias assessment for the included studies

The evaluation of potential bias was carried out for the thirteen included case-control studies using the NOS tool (Table 4). The overall quality of the included studies could be described as having a high risk of bias based on NOS assessment. All studies were showing high risk of bias owing to poor study design and lack of sample size justification and ascertainment of exposure, with the scores ranging from 2 to 6. A relatively low risk was observed for the questions ‘Representativeness of the cases’ (10/13) and ‘Comparability between cases and controls’ (10/13). While, a high risk of bias was observed for most of the studies in terms of inadequate case definition (7/13), Selection bias (3/13), Definition of Controls (4/13), Ascertainment of exposure (2/13), Same method of ascertainment for cases and controls (1/13). The results of the ROB assessment is presented in the Table 4.

**Table 4:**
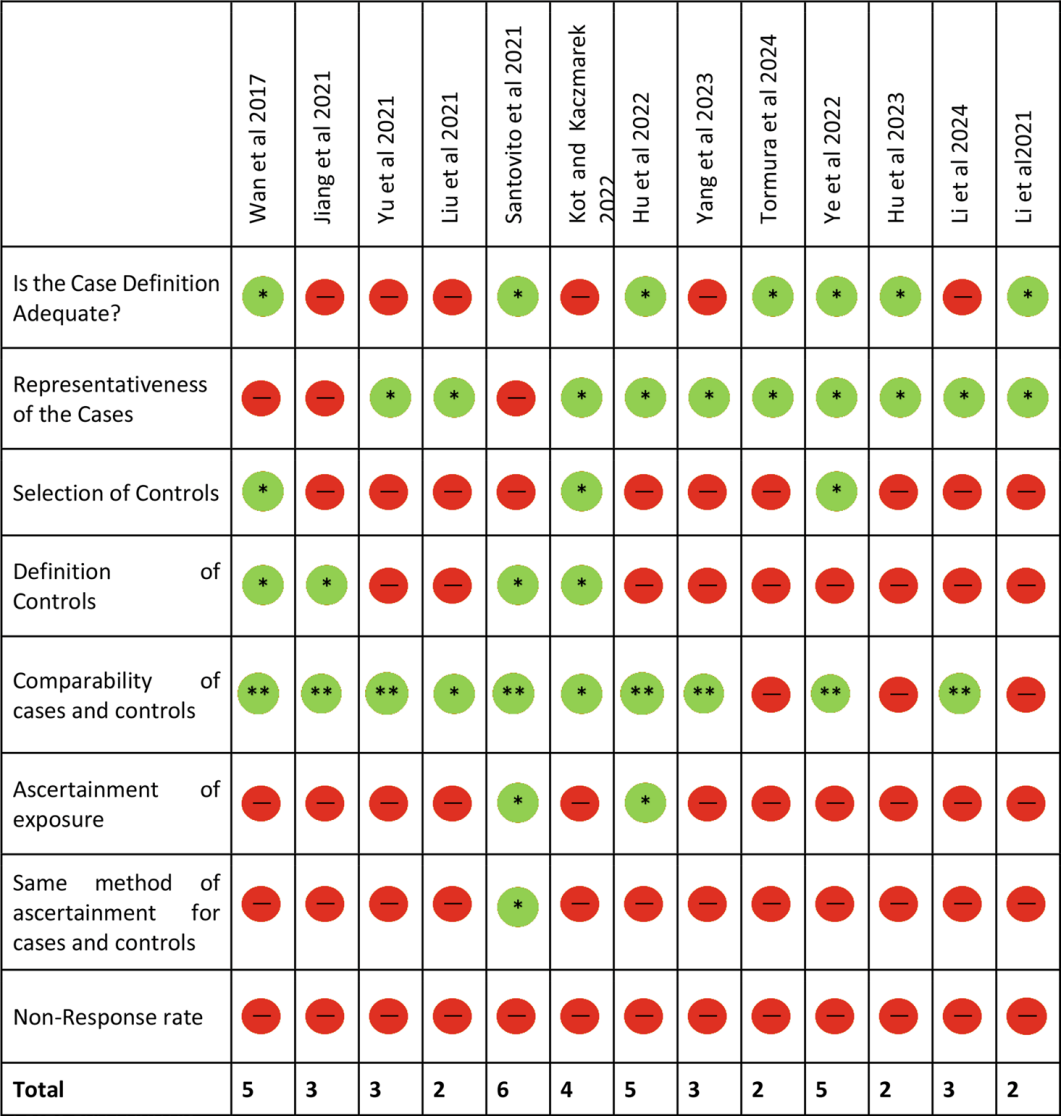
Risk of bias assessment of the included studies using Newcastle – Ottawa quality assessment scale

## Discussion

Early diagnosis of DR is critical to facilitate early intervention which can prevent disease progression and vision loss. Recently, non-coding RNAs have emerged as potential diagnostic markers for various diseases including DR [39]. Several research papers have analysed the role of ncRNAs, particularly of exosomal origin in regulating the development and progression of DR [40, 41]. In this systematic review, we synthesised data from thirteen human studies that identified the expression and role of exosomal regulatory ncRNAs in individuals with DR. We qualitatively synthesised the ncRNAs expression patterns and their potential involvement in pathogenic pathways associated with DR. However, we did not identify any common differentially expressed ncRNAs across the studies, raising questions about the robustness and generalizability of the findings. The lack of experimental validation of these ncRNA in the existing literature also underscores the potential constraints in the present comprehension of exosomal RNA’s role in DR. Thus, the current body of evidence is considered insufficient to attain a conclusive understanding of the causal relationship of exosomal non-coding RNAs as a biomarker for diabetic retinopathy.

Understanding the precise mechanisms driving the progression of ocular diseases is imperative for developing targeted drugs. Examining the differences in exosomal ncRNA cargo between healthy and affected individuals may reveal key pathways in DR. The classification of non-coding RNAs into various categories is still in its nascent stages of comprehension. While the majority of the research till date has focused on miRNAs, the role of other forms like lncRNA, and circRNA is only now getting recognized. In our systematic review, we observed the investigation of different types of exosomal ncRNAs, with nine studies emphasising miRNAs, three on lncRNAs, and only one on circRNA.

Our systematic review identified 13 studies that presented exosomal ncRNA expression profiles in individuals with DR. However, none of these miRNAs exhibited consistent differential expression across the investigations. One possible rationale could be the heterogeneity in the biological sources of these ncRNAs. Among the nine investigations, two utilised plasma, four utilized serum, four utilized vitreous humor, and two utilized tear samples. This variance might indicate unique functions fulfilled by these ncRNAs at distinct anatomical sites. Nevertheless, this conjecture necessitates validation in forthcoming investigations that juxtapose non-coding RNA expression in various sample types for DR. In the majority of the investigations, samples were pooled; this practice is also deemed suboptimal as it has the potential to distort the outcomes. The mechanistic details of how these differentially expressed ncRNAs are involved in the development and progression of DR will have to be investigated in more detail in future studies.

**Fig. 2:**
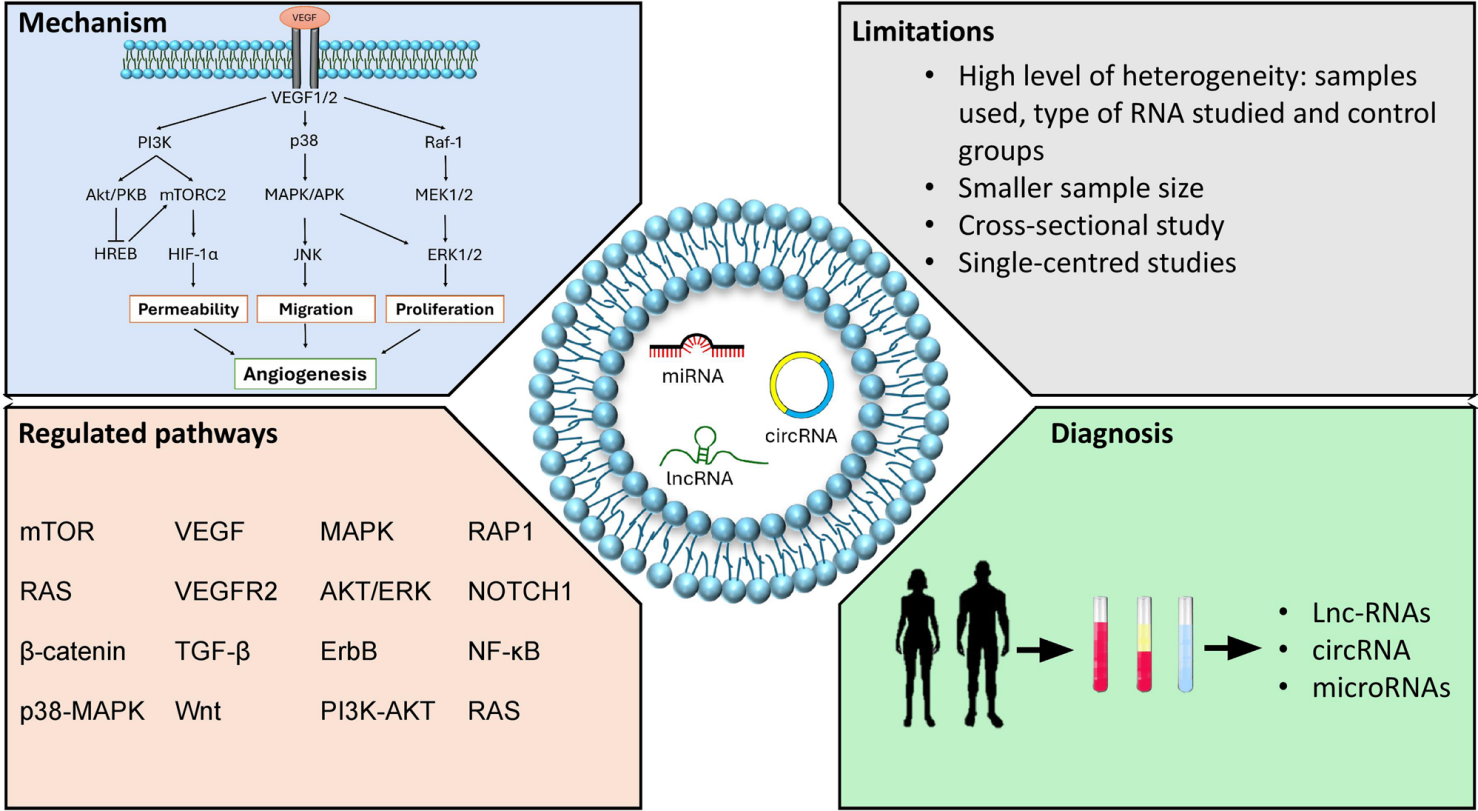
Use of exosomal non-coding RNAs (ncRNAs) as biomarkers for Diabetic Retinopathy (DR). Top left shows the central mechanism for DR progression; Bottom left lists the key pathways through which ncRNAs regulate DR; Top right describes the limitations of the studies included in this work; Bottom right depicts how ncRNAs from human samples can be used for early diagnosis of DR

Alongside profiling, exploration of regulatory pathways associated with these dysregulated ncRNA is crucial to understanding their role in DR pathology [42, 43]. Several studies have employed various bioinformatics tools such as TargetScan, GO, KEGG, and miRTarBase to identify and analyse target genes and pathways regulated by differentially expressed exosomal ncRNAs. As a result, various signalling pathways that regulate angiogenesis, apoptosis, inflammation, and cell proliferation have been linked to the differentially expressed ncRNAs in our included studies. Jiang et al., Kot et al., Liu et al., and Li et al., [25–27, 33] have indicated that ncRNAs regulate the VEGF signalling pathway, while Yu et al., Ye et al., and Li et al. [32, 34, 35] demonstrated that ncRNAs regulate the mitogen-activated protein kinase (MAPK) cell signalling pathway. Two studies Yu et al., and Li et al. [32, 35] demonstrated that upregulated ncRNAs regulates through RAS pathway, whereas, Kot and Kaczmarek, and Ye et al. [26, 34] have demonstrated the regulation of TGF-β. In brief, although these studies identified an atypical patterns of exosomal ncRNAs derived from patient samples and their computational analysis in comprehending the molecular pathways implicated in DR pathogenesis (Fig. 2), further in vitro and in vivo validation is crucial to confirm these findings and fully elucidate the enigmatic roles of exosomal ncRNAs.

Our systematic review focusses on exosomes as the source for ncRNAs. It is proposed that they have greater advantages as diagnostic and prognostic biomarkers compared to traditional test methods such as circulating tumour cells (CTCs) and circulating tumour DNA (ctDNA) [44]. However, research on exosomal ncRNAs particularly in DR is limited. Several studies have examined non-exosomal circulating ncRNAs and demonstrated their involvement in the pathological processes of various diseases [45], including DR [46, 47]. One study highlighted that non-exosomal miRNAs were more upregulated compared to exosomal miRNAs [30]. More research is needed to determine whether exomiRs are better than free circulating miRNAs. A study by Kamal and Shahidan (2020) [17], pointed out that 71% of the studies screened from 69 articles, recommended exosomal miRNAs over non-exosomal miRNAs. While there is a multitude of evidence suggesting the involvement of exosomal ncRNAs in the development of diabetic complications through various molecular mechanisms [48], definitive conclusions have not been reached. Further investigations are warranted to explore their potential utility as innovative biomarkers for timely detection, disease surveillance, and evaluation of treatment efficacy. It is imperative for forthcoming studies to embrace a cohesive methodology encompassing suitable research frameworks, sample dimensions, and rigorous distinctions between exosomal and exosome-depleted ncRNAs derived from identical subjects and compared appropriately between the case and controls.

Our systematic review has certain limitations, mainly stemming from the lack of sufficient primary studies on exosomal RNA expression. Firstly, due to lack of comparable quantitative data, we could only adapt a qualitative approach to synthesise the results of the individuals studies. Secondly, the studies exhibited a high level of heterogeneity in terms of samples used, types of RNA included, and the control groups involved. Thirdly, the population of patients with DR in our study included only a few patients with proliferative-stage retinopathy, and thus, we were not able explore differences in the exosomal ncRNAs between patients at different stages of DR. Fourthly, given that these studies are case-control, we cannot clarify causal associations between the identified exosomal ncRNAs and DR. Lastly, the single-centre nature of the data source may have introduced selection bias. Also, the lack of data regarding age, gender, and comorbidities could potentially impact the outcome. Lastly, the selected studies used different techniques to isolate and quantify ncRNAs, which becomes a source of variability and makes it impossible to perform a reliable quantitative analysis of the data (meta-analysis).

## Conclusion

In conclusion, this systematic review provides a comprehensive overview of the current research landscape on exosomal ncRNA expression as a diagnostic marker for early detection of DR. Our review sheds light on the identified biomarkers that have the potential to be developed for early diagnosis of DR. We also effectively identified a knowledge gap in the existing literature, emphasizing the need for future research to address the methodological variations in the studies and enhance the reliability of results.

## Electronic supplementary material

Below is the link to the electronic supplementary material.

Supplementary Material 1

## Data Availability

No datasets were generated or analysed during the current study.

## Abbreviations

ACY-1: Aminoacylase-1
ARHGAP: Rho GTPase activating protein
circRNAs: Circular RNAs
CTC: Circulating tumour cells
CtDNA: Circulating tumour DNA
DE: Differentially expressed
DEGs: Differentially expressed Genes
DLX6-AS1: Distal-less homeobox 6 antisense 1
DM: Diabetes mellitus
DME: Diabetic macular edema
DR: Diabetic Retinopathy
FAM190A-3: Family with sequence similarity 190, member A3
lncRNAs: Long noncoding RNAs
MAPK: Mitogen-activated protein kinase
miRNAs: MicroRNAs
MH: Macular hole
ncRNA: Noncoding RNAs
NOS: Newcastle- Ottawa Scale
PDR: Proliferative diabetic retinopathy
PI3K: Phosphatidylinositol 3-kinase
PRINS: Psoriasis-susceptibility-related RNA gene induced by stress
PRISMA: Preferred reporting items of systematic reviews and meta-analysis
RAF-1: Rapidly accelerated fibrosarcoma-1
T1D: Type 1 diabetes
T2DM: Type 2 diabetes
TRD: Tractional retinal detachment
VEGF: Vascular endothelial growth factor
